# Parasites of importance for human health in Nigerian dogs: high prevalence and limited knowledge of pet owners

**DOI:** 10.1186/1746-6148-4-49

**Published:** 2008-12-09

**Authors:** Uade Samuel Ugbomoiko, Liana Ariza, Jorg Heukelbach

**Affiliations:** 1 Department of Zoology, University of Ilorin, Nigeria; 2 Post-Graduation Program in Medical Sciences, School of Medicine, Federal University of Ceará, Brazil; 3 Department of Community Health, School of Medicine, Federal University of Ceará, Rua Prof. Costa Mendes 1608, 5. andar, Fortaleza CE 60430-140, Brazil; 4 Anton Breinl Centre for Public Health and Tropical Medicine, School of Public Health, Tropical Medicine and Rehabilitation Sciences, James Cook University, Townsville, Australia

## Abstract

**Background:**

Dogs are the most common pet animals worldwide. They may harbour a wide range of parasites with zoonotic potential, thus causing a health risk to humans. In Nigeria, epidemiological knowledge on these parasites is limited.

**Methods:**

In a community-based study, we examined 396 dogs in urban and rural areas of Ilorin (Kwara State, Central Nigeria) for ectoparasites and intestinal helminths. In addition, a questionnaire regarding knowledge and practices was applied to pet owners.

**Results:**

Nine ectoparasite species belonging to four taxa and six intestinal helminth species were identified: fleas (*Ctenocephalides canis, Pulex irritans, Tunga penetrans*), mites (*Demodex canis, Otodectes *sp., *Sarcoptes scabiei *var. *canis*), ticks (*Rhipicephalus sanguineus, Ixodes *sp.), and lice (*Trichodectes canis*); and *Toxocara canis, Ancylostoma *sp., *Trichuris vulpis, Dipylidium caninum*, Taenidae and *Strongyloides *sp. Overall prevalence of ectoparasites was 60.4% and of intestinal helminths 68.4%. The occurrence of *C. canis, R. sanguineus, T. canis, Ancylostoma *sp. and *T. vulpis *was most common (prevalence 14.4% to 41.7%). Prevalence patterns in helminths were age-dependent, with *T. canis *showing a decreasing prevalence with age of host, and a reverse trend in other parasite species. Knowledge regarding zoonoses was very limited and the diseases not considered a major health problem. Treatment with antiparasitic drugs was more frequent in urban areas.

**Conclusion:**

Parasites of importance for human health were highly prevalent in Nigerian dogs. Interventions should include health education provided to dog owners and the establishment of a program focusing on zoonotic diseases.

## Background

Dogs are the most successful canids, adapted to human habitation worldwide. They have contributed to physical, social and emotional well-being of their owners, particularly children [1,2]. However, in spite of the beneficial effects, close bonds of dogs and humans (in combination with inappropriate human practices and behaviour) remain a major threat to public health, with dogs harbouring a bewildering number of infective stages of parasites transmissible to man and other domestic animals [2-4]. For example, well-known and important zoonotic diseases are cutaneous and visceral larva migrans, hydatid disease and tungiasis [5-8].

In low-income settings, treatments to eliminate these parasites are – if done at all - often applied in advanced stages of disease, causing distress on pets and their owners [9,10].

In many African countries, including Nigeria, appropriate policies regarding pet ownership and their effects on individual and community health are nonexistent. Prevalence of parasite infection in dogs with importance for human health is usually high, resulting in risk of zoonotic transmission from dogs to humans. The risk is further increased by non-favourable ecological and human behavioural factors [11-13].

Previous epidemiological studies on dog parasites in Nigeria were focused on the prevalence with little or no information on quantitative measure of infection and/or were not community-based [14-17]. Thus, we examined a representative population of dogs in urban and rural areas in a Nigerian city for the presence of possibly zoonotic parasites.

## Methods

### Study Area

**Figure 1:**
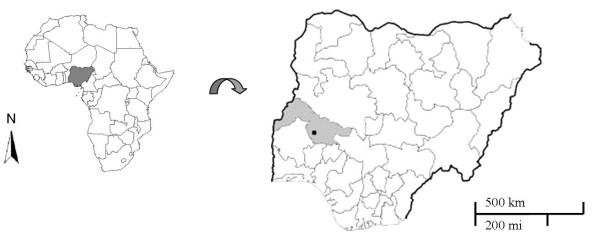
Map of the study area highlighting Nigeria (left), Kwara State and the study area (right).

The study was conducted in the city of Ilorin (Central Nigeria), and the neighbouring rural communities (longitude 4° 30' – 4° 45'N and latitude 8° 28' – 8° 38'E; Figure 1). Ilorin is an urban centre and the capital of Kwara State. The city covers an area of about 38 square miles, with an estimated population of 1.4 million people. It is located in Nigeria's central savannah region with intense rainfalls from April to October and daily temperatures between 23°C and 37°C.

The urban area of Ilorin is surrounded by rural villages with mainly agricultural-based economy. Living conditions are particularly poor in these rural communities, and a substantial proportion of the villagers keeping dogs have no access to veterinary services. Therefore, most dogs have never been treated for any form of parasitic diseases prior to the study. In addition, most dogs are not vaccinated.

### Study design

A random house-to-house screening of dogs was conducted between October 2006 and May 2007. With the informed consent of dog owners, interviews were conducted using pre-tested structured questionnaires to obtain information on the dogs' age, sex, regimen, defaecation sites, previous anthelminthic treatment and disease-related knowledge of owners. Thereafter, pre-labeled specimen containers were distributed for the collection of stool samples. A screening of ectoparasites on dogs was performed before fecal specimen collection.

In households with more than one dog, only one dog (chosen by the dog owner) was included.

### Sample collections

Dogs were thoroughly examined for ectoparasites by combing the entire body surfaces on a clear sheet of white paper. To facilitate the extraction of ectoparasites, the dogs were rubbed with a piece of cotton-wool soaked in ether. The ectoparasites recovered were preserved in 70% alcohol for identification.

For the diagnosis of intestinal helminths, freshly passed faecal samples from dogs were collected from dog owners and examined macroscopically for proglottides. Thereafter, a sub-sample of faeces was taken into a pre-labelled clean sterile universal plastic bottle containing 10% formaldehyde solution. All samples were carried to the parasitology laboratory at the University of Ilorin and processed for microscopic examination.

### Laboratory procedures

Fleas, ticks and lice were cleared in 10% potassium hydroxide (KOH) solution overnight, dehydrated in ascending strength of alcohol and mounted in Canada balsam. Mites were mounted directly in Berlese fluid. Examination was done at 40× magnification under a dissecting microscope.

A duplicate 50 mg Kato-Katz thick smear was prepared from each faecal sample, using the Kato-Katz technique, as modified by Forrester and Scott [18]. In short, a small portion (1–3 g) was sieved through double-ply gauze to remove rough materials. The filtrate was centrifuged at 3000 rpm for 3 min, the supernatant decanted, and the tube allowed to stand for 10 min. Fifty mg of the sediment delivered by Kato-Katz template was taken onto a degreased glass slide, and covered with a cellophane strip soaked overnight in 50% solution of glycerol-malachite green. Slides were examined for helminths eggs under a light microscope immediately after preparation. Parasite eggs were identified based on the morphological characteristics. Density of infection, as expressed by eggs per gram (EPG) of faeces, was calculated by multiplying each slide count by 20 [19].

### Data Entry and Statistical Analysis

Data were entered using an excel spreadsheet and checked for entry errors, by comparing data entries with the original data forms. Then, data were transferred to Stata^® ^software package (version 9.0; Stata Corporation, College Station, USA) for analysis. The Fisher's exact test was applied to determine the significance of differences of relative frequencies and the one-way ANOVA test to determine significance of differences of mean egg counts.

## Results

A total of 396 dogs, consisting of 180 (45.5%) males and 216 (54.5%) females was examined; 192 (48%) dogs lived in urban, and 204 (52%) in rural areas.

All dog owners agreed to participate and completed the questionnaires. Table 1 summarizes the differences in dog regimen and the perception of dog owners to diseases transmissible by their animals, stratified by urban and rural areas. In the rural area, significantly more individuals kept dogs for hunting and observed their dogs catching prey than in the city (*p *< 0.0001), whereas 29.2% and 18.1% of dog owners in the urban and rural areas kept dogs as watch dogs, respectively (Table 1). Treatment with antiparasitic drugs was a more frequent practice for dogs from urban than rural areas.

**Table 1:** Characteristics of dogs and knowledge and attitudes of dog owners regarding potential zoonotic disease in urban and rural communities.

Variables	Urban n = 192		Rural n = 204		Urban Vs. rural
			
	N	%	N	%	*p *value
**Sex of dogs**					
Male	91	47.4%	89	43.6%	0.5
Female	101	52.6%	115	56.4%	0.5
**Age of dogs (months)**					
0–6	61	31.8%	73	35.8%	0.4
7–11	49	25.5%	59	28.9%	0.4
≥ 12	82	42.7%	72	35.3%	0.1
**Reasons for keeping dogs**					
Hunting	71	37.0	115	56.4	< 0.0001
Watch dog	56	29.2	37	18.1	0.013
Companion	43	22.4	32	15.7	0.1
No specific reason	22	11.5	20	9.8	0.6
**Where do dogs usually roam?**					
Confined to dog house on compound	18	9.4	7	3.4	0.022
Inside the house	3	1.6	11	5.4	0.055
Within the compound	57	29.7	33	16.2	0.002
Anywhere within and outside the compound	114	59.4	153	75.0	0.001
**How do dogs leave house premises**					
Always accompanied	35	18.2	10	4.9	< 0.0001
Occasionally accompanied	67	34.9	80	39.2	0.4
Never Accompanied	90	46.9	114	55.9	0.09
**Usual place of defecation**					
Within the house premises	66	34.4	54	26.5	0.1
Within/out of house premises	126	65.6	150	73.5	0.1
**Preferred type of floor where dogs defecate**					
Only impervious (cemented/tiles)	29	15.1	16	7.8	0.027
Only pervious (grass, soil, etc)	56	29.2	86	42.2	0.009
Both pervious/impervious	107	55.7	102	50.0	0.3
**Observation on dogs catching prey**	118	61.5	164	80.4	< 0.0001
**Last anthelminthic treatment of dogs**					
< 12 months ago	42	21.9	20	9.8	0.001
≥ 12 months ago	56	29.2	38	18.6	0.018
Never	94	49.0	146	71.6	< 0.0001
Dog owners' knowledge of possible diseases/conditions transmitted or caused by dogs*					
Rabies	124	64.6	136	66.7	0.7
Wound from dog bite	75	39.1	85	41.7	0.6
Scabies	35	18.2	28	13.7	0.3
Worms	11	5.7	15	7.4	0.5
Dysentery	6	3.1	10	4.9	0.4
Other bacterial/viral diseases	5	2.6	12	5.9	0.14
**Do children play with dogs?**					
Yes	191	99.5	204	100	0.5
No	1	0.5	0	0	0.5
**Dog owners' perception of diseases transmitted by dogs**					
Serious	35	18.2	23	11.3	0.064
Not serious	91	47.4	72	35.3	0.019
Do not cause any disease	66	34.4	109	53.4	< 0.0001

*more than one option possible

Interestingly, more than half of dog owners in the rural communities, and about a third in the urban area did not perceive diseases transmitted by dogs as a health problem (*p *< 0.0001). The bonds of humans with their animals were close, and children played with virtually all dogs included in the study (Table 1). When asked about possible diseases transmitted by their dogs, less than 10% of owners mentioned helminths ("worms") as a health problem, but about two third were aware of the risk of rabies transmission (Table 1).

### Ectoparasites

At least one of nine ectoparasite species identified, belonging to four taxa, was encountered in 239 (60.4%) of the 396 dogs. Dogs from rural areas (77.9%) were more commonly infested than those from urban areas (41.7%; *p *< 0.0001). Eighty (20.3%) dogs harboured two or more species. Dogs from rural areas were more frequently parasitized with two or more ectoparasites than the urban dogs (Table 2).

**Table 2:** Prevalence of ectoparasites in dogs, stratified by urban or rural communities.

	Overall (n = 396)		Urban (n = 192)		Rural (n = 204)		Urban vs. Rural
	N infected	% (95%CI)	N infected	% (95%CI)	N infected	%(95%CI)	*p *value
**Fleas**							
*Ctenocephalides canis*	127	32.1(27.5 – 6.9)	40	20.8(14.9 – 26.7)	87	42.6(35.8 – 49.7)	< 0.0001
*Pulex irritans*	26	6.6(4.3 – 9.5)	7	3.6(1.5 – 7.4)	19	9.3(5.7 – 14.2)	0.026
*Tunga penetrans*	2	0.5 (0.0 – 1.8)	0	0.0	2	1.0(0.0 – 3.5)	0.5
**Mites**							
*Demodex canis*	4	1.0 (0.0 – 2.6)	0	0.0	4	2.0(0.0 – 4.9)	0.12
*Otodectes *sp.	39	9.8(7.1 – 13.2)	6	3.1(1.2 – 6.7)	33	16.2(11.4 – 22.0)	< 0.0001
*Sarcoptes scabiei var. canis*	8	2.0(0.1 – 3.4)	1	0.5(0.0 – 2.9)	7	3.4(1.3 – 6.9)	0.069
**Ticks**							
*Rhipicephalus sanguineus*	76	19.2(15.4 – 23.4)	25	13.0(8.6 – 18.6)	51	25.0(19.2 – 31.5)	0.003
*Ixodes *sp.	18	4.5(2.7 – 7.1)	7	3.6(1.5 – 7.4)	11	5.4(2.7 – 9.4)	0.5
**Lice**							
*Trichodectes canis*	42	10.6(7.8 – 14.1)	15	7.8(4.4 – 12.6)	27	13.2(8.9 – 18.7)	0.1
**Number of ectoparasite species per host**							
One ectoparasite species	159	40.2(35.3 – 45.2)	60	31.3(24.8 – 38.3)	99	48.5(41.5 – 55.6)	< 0.0001
Two ectoparasite species	72	18.2(14.5 – 22.3)	19	9.9(6.1 – 15.0)	53	26.0(20.1 – 32.6)	< 0.0001
Three ectoparasite species	7	1.8(0.7 – 3.6)	1	0.5(0.0 – 2.9)	6	2.9(1.1 – 6.3)	0.12
Four or more ectoparasite species	1	0.3(0.0 – 1.4)	0	0.0	1	0.5(0.0 – 2.7)	1.0

In total, 155 (39.1%) were infested with fleas, 94 (23.7%) with ticks, 51 (12.9%) with mites, and 42 (10.6%) with lice. The prevalence detailed for each ectoparasite species is depicted in Table 2, stratified by urban and rural areas.

The common dog flea, *Ctenocephalides canis*, was the most prevalent species and present in almost one third of dogs, followed by the brown dog tick *Rhipicephalus sanguineus*, *Trichodectes canis*, *Otodectes *sp., *Pulex irritans *and *Ixodes *sp. (Table 2). Infestations due to the sand flea *Tunga penetrans*, the mange mite *Sarcoptes scabiei *var. *canis *and *Demodex canis *were less common.

The prevalence of *C. canis *and of *Otodectes *sp. was significantly higher in rural dogs than in urban dogs. A similar trend was observed for *P. irritans *and *R. sanguineus *(Table 2).

### Intestinal helminths

In total, 271 (68.4%) of the examined dogs were infected with at least one intestinal helminth species. Six species, namely *Toxocara canis, Ancylostoma *sp.*Trichuris vulpis, Dipylidium caninum*, Taenidae and *Strongyloides *sp. were identified in dogs of both urban and rural areas (Table 3).

**Table 3:** Prevalence of intestinal helminths parasite in dogs, stratified by communities.

	Overall (n = 396)		Urban (n = 192)		Rural (n = 204)		Urban vs. Rural
	N infected	% (95% Cl)	N infected	% (95% Cl)	N infected	% (95% Cl)	*p *value
**Parasite species**							
*Toxocara canis*	165	41.7 (36.8 – 46.7)	72	37.5 (30.6 – 44.8)	93	45.6 (39.6 – 53.7)	0.13
*Ancylostoma *sp.	67	16.9 (13.4 – 21.0)	27	14.1 (9.5 – 19.8)	40	19.6 (14.4 – 25.7)	0.18
*Trichuris vulpis*	57	14.4 (9.7 – 16.6)	28	14.6 (9.9 – 20.4)	29	14.2 (9.7 – 19.8)	1.0
*Dipylidium caninum*	36	9.1 (6.5 – 12.4)	11	5.7 (2.9 – 10.0)	25	12.3 (8.1 – 17.6)	0.035
Taenidae	33	8.3 (5.8 – 11.5)	3	1.6 (0.3 – 4.5)	30	14.7 (10.2 – 20.3)	< 0.0001
*Strongyloides *sp.	15	3.8 (2.1 – 6.2)	3	1.6 (0.3 – 4.5)	12	5.8 (3.1 – 10.1)	0.033
**Number of intestinal helminth species per host**							
One helminth species	196	49.4 (44.2 – 54.3)	92	47.9 (40.7 – 55.2)	104	51.0 (43.9 – 58.3)	0.6
Two helminth species	52	13.1 (10.0 – 16.9)	17	8.9 (5.2 – 13.8)	35	17.5 (12.3 – 23.0)	0.017
Three helminth species	18	4.6 (2.7 – 7.1)	4	2.1 (0.6 – 5.2)	14	6.9 (3.8 – 11.2)	0.029
Four or more helminth species	5	1.3 (0.0 – 2.9)	0	-	5	2.5 (0.1 – 5.6)	0.062

The most common parasites were *T. canis*, followed by *Ancylostoma *sp. and *T. vulpis *(Table 3). Prevalence of the dog tapeworm, *D. caninum*, Taenidae and *Strongyloides *sp. were <10%.

Except for *D. caninum*, Taenidae and *Strongyloides *sp., the prevalence of intestinal helminths was not statistically different in urban or rural areas. However, multiple infections with 2 and 3 parasites species per host were significantly higher in rural than in urban areas (Table 3).

**Figure 2:**
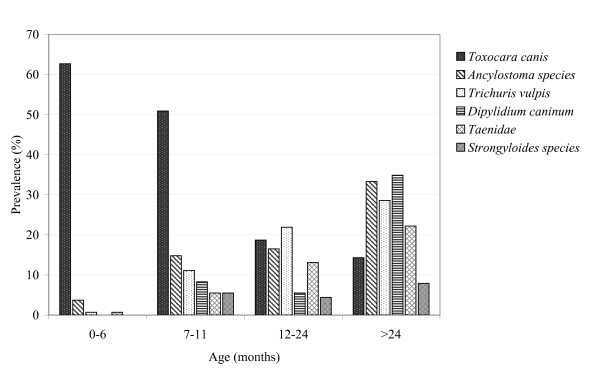
Prevalence of intestinal helminths species diagnosed in dogs, stratified by age of dogs.

The pattern of prevalence and distribution of helminth parasites, stratified by age of dogs, is depicted in Figure 2. In general, prevalence of parasite infection increased with age of the dog. An exception was observed in *T. canis *infection, which was by far the most common infection in puppies, and showed decreasing prevalence with age. The density of infection, expressed by mean egg counts per gramme (epg) paralleled the prevalence data (Table 4).

**Table 4:** Density of intestinal parasites infection in dogs, stratified by rural and urban communities.

Parasite	Overall n = 396	Urban n = 192	Rural n = 204	Urban vs. Rural
	
	Mean (SD)	Mean (SD)	Mean (SD)	*p *value
*Toxocara canis*	375.6 (569.5)	264.1 (441.3)	480.47 (651.9)	0.001
*Ancylostoma *sp.	84.2 (221.5)	70.4 (207.6)	97.29 (233.5)	0.54
*Trichuris vulpis*	147.8 (440.6)	126.23 (404.2)	168.12 (477.4)	0.79
*Dipylidium caninum*	46.8 (189.7)	10.12 (85.9)	81.29 (246.2)	0.028
Taenidae	126.8 (435.0)	77.38 (336.6)	173.29 (507.1)	0.001
*Strongyloides *sp.	11.3 (68.1)	7.25 (57.8)	15.18 (76.5)	0.92

## Discussion

The present study provides the first systematic assessment on quantitative estimates of parasites in dogs in Nigeria's Kwara State. The results show that ectoparasitic and intestinal helminth species were abundant, and that prevalence and density of infection was very high. The knowledge and perception of dog owners regarding zoonotic diseases transmitted by pets was insufficient.

The parasites reported in this study have been previously documented in dogs throughout the world, with a pronounced difference in prevalence and density between regions [16,17,20-27]. In our study, the overall prevalence of intestinal helminths (68%) was similar to that reported from different ecological and epidemiological settings in Nigeria [17,26] and to the prevalence of 71% reported from Spain [28]. In South Africa (76%), Mexico (85%) and Morocco (100%), prevalences were even higher [22,23,29].

This potential for human zoonotic disease has rarely been addressed in control programs in Nigeria and other low income countries. Considering the high prevalence of ectoparasites and intestinal helminth infections found in dogs, and the close bonds in which dogs live together with people, the risk of transmission of these parasites to humans seems to be obvious. For example, *Toxocara *infection in humans may cause visceral larva migrans, in severe cases leading to blindness [30], and dog hookworm infections put humans at risk for cutaneous larva migrans which is endemic in many resource-poor communities [31]. *Rhipicephalus *ticks have been described to parasitize humans [32], and may transmit rickettsial disease and visceral leishmaniasis [33]. Fleas may transmit human plague, rickettsioses and trypanosomes [34], and serve as intermediate hosts for the dog tapeworm, *D. caninum*.

Our data show that the prevalence pattern was age-dependent; *T. canis *decreased with age of dog, whereas *A. caninum, T. vulpis*, Taenidae, *D. caninum *and *Strongyloides *sp. increased with age, even though to a less extent. These patterns have been observed previously [16,17,20,23,27]. In Nigeria, Sowemimo and Asaolu [27] found by far the highest prevalence of toxocariasis in puppies, whereas the age dependency of hookworm infection was less pronounced. The high prevalence of ascarid infections in puppies is in accordance with the transmission pattern of the parasite, which is mainly by transplancental and transmammary routes; acquired age-dependent immunity may be caused by repeated exposure [35,36]. Increased infection rates in older dogs are caused by parasite species which are not transmitted to dogs at early age, and thus do not elicit a specific immune response.

The prevalence detected in our study differs from those of Sowemimo and Asaolu [27] who recorded 24% in a Nigerian city in a neighbouring state with similar characteristics as Ilorin. However, these data were not population-based, but included dogs presented to veterinary clinics. These authors also argued that the reduction of prevalence as compared to a study done in the 1970s [31] may be caused by increased awareness of pet holders regarding deworming practices. In contrast, our data can be regarded as representative for the dog population, as pet owners who bring their animals to veterinary clinics may deworm their animals more regularly. As a consequence, studies based on veterinary clinics underestimate prevalence of parasitic infections and infestations. Our data, though, show that the majority of dogs received antiparasitic treatment never or more than a year ago, and only few people were aware of the zoonotic potential of dog parasites; 60% of dogs examined had never visited a clinic for any form of treatment.

The reduced prevalence of *D. caninum *over time was claimed to be caused by the reduced prevalence of the intermediate host *C. canis*. This may hold true for pets brought to veterinary clinics, but our study shows that *C. canis *is very common in dogs in the community and thus probably continue being important for the transmission of *D. caninum*.

The intensities of *T. canis*, Taenidae and *D. caninum *were statistically higher in rural dogs than those in the urban area. Similarly, Habluetzel et al. [38] observed that twice as many dogs from rural areas had nematodes infections, as compared to urban dogs. These differences in the level of infection from different locations have been described also in other studies [39,40] and may be partly due to variation in local environmental conditions affecting spatial aggregation and infective stages of parasites. Besides, differences in health care and animal management practices may account for these differing characteristics. Urban dog owners may feel encouraged by their proximity to veterinary clinics, which are nonexistent in rural areas.

The number of intestinal parasite species per host revealed that single infection was more common; polyparasitism with more than two parasites species was less frequently observed. A similar pattern was observed in ectoparasite infestation. These results are in agreement with Fontanarrosa et al. [24] who explained that interactions among parasite species depend on parasite burden rather than the mere presence of other species.

The high prevalence of ectoparasites (60%) was consistent with another study, where fleas and ticks were the most commonly found taxa [41]. Ugochukwu and Nnadozie [42] recorded in Bendel State (Nigeria) a low prevalence of ectoparasites in dogs, including *Demodex canis, R. sanguineus *and *C. canis*. Bryson et al. [43] identified several species of ixodid ticks, fleas and lice from dogs in South Africa. However, *C. felis *and *Echidnophaga gallinacea *which were frequently reported in dogs in other study areas [39,42-44] were not encountered in our study.

The variation in distribution and prevalence of ectoparasites can be ascribed to differences in the availability of infective stages, host habitat/climatic factors and the sampling period. Peak prevalences of ectoparasites usually occur during the warm dry months [40,45,46]. Gracia et al. [40] revealed that accumulation of organic wastes and the presence of other pet animals influence the survival and abundance of ectoparasites, especially fleas. This also explains why *P. irritans *and *T. penetrans*, relatively low host-specific ectoparasites, occurred only in rural areas, where dogs were frequently in contact with other natural host animals, such as pigs, rats and small ruminants [47-49].

Unfortunately, due to the absence of funding, we were unable to identify the prevalence of other zoonotic diseases and to specify the species in Taenidae encountered, such as *Echinococcous granulosus *causing hydatid disease. The diagnostic technique of parasites done in this study, based on the morphological characteristics of ova under light microscope, has the disadvantage that it fails to distinguish *E. granulosus *from other Taenidae. Thus, *E. granulosus*, a major zoonotic parasite of livestock and dogs in Nigeria [11,14,15], has possibly been present but not reported in our survey. The fact that dogs enjoy unrestrained association with humans, scavenge for food in an environment contaminated with faecal material of potential intermediate hosts and feed on offal of slaughtered livestock in abattoirs (Ugbomoiko, personal communication) makes transmission of hydatid disease predictable in the setting studied.

In general, the trend in prevalence, density and species composition of parasites observed in this study may reflect the degree of environmental contamination and inequalities in the health care service between urban and rural areas. In particular, *T. canis*, *A. caninum *and *D. caninum *are zoonotic parasites constituting public health problems in the study areas.

## Conclusion

Our study shows that parasites of importance for human health were highly prevalent in Nigerian dogs and that intervention measures are necessary to reduce the risk of transmission of parasites from dogs to humans. Interventions should focus on health education provided to dog owners and the establishment of a program based on zoonotic diseases.

## Authors' contributions

USU: study design, conducted the study, statistical analysis, contributed to the manuscript; LA: statistical analysis, contributed to the manuscript; JH: study design, statistical analysis, contributed to the manuscript. All authors read and approved the final manuscript.
